# Editorial: Community series in immunometabolic mechanisms underlying the severity of COVID-19, volume II

**DOI:** 10.3389/fimmu.2023.1221642

**Published:** 2023-06-01

**Authors:** Julia Kzhyshkowska, Vishwanath Venketaraman, Galileo Escobedo

**Affiliations:** ^1^ Institute of Transfusion Medicine and Immunology, Institute for Innate Immunoscience (MI3), Medical Faculty Mannheim, Heidelberg University, Mannheim, Germany; ^2^ German Red Cross Blood Service Baden-Württemberg - Hessen, Mannheim, Germany; ^3^ National Research Tomsk State University, Tomsk, Russia; ^4^ Department of Basic Medical Sciences, College of Osteopathic Medicine of the Pacific, Western University of Health Sciences, Pomona, CA, United States; ^5^ Laboratory of Immunometabolism, Research Division, General Hospital of Mexico “Dr. Eduardo Liceaga”, Mexico City, Mexico

**Keywords:** SARS-CoV-2, COVID-19, inflammation, macrophage, neurodegeneration, aminoacid, virus

The contribution of metabolic diseases to the severity of the acute inflammatory reaction against the severe acute respiratory syndrome coronavirus-2 (SARS-CoV-2) and multiorgan damage has been demonstrated by many cohort studies from diverse geographic areas and ethnic origins (1). However, the mechanism of how SARS-CoV-2 can change immune-metabolism, both local and systemic, is insufficiently understood since most studies analyzing the acute phase have been focused on the inflammatory profiles, cytokine storm, and organ-specific pathological reactions. Volume II of the Research Topic entitled “*Immunometabolic Mechanisms Underlying the Severity of COVID-19*” is dedicated to making links between SARS-CoV-2-mediated changes in systemic metabolism and to placing such changes in the context of a complex interaction between inflammatory and neurodegenerative pathways, both highly characteristic for acute and long-COVID-19 sequelae (2).

The focus on the innate immune responses was justified by the study by Zhu et al., who applied machine learning to process flow cytometry data sets containing the peripheral blood information of COVID-19 patients. The input features included immune cell counts and the activation marker concentrations of specific cells. The developed model suggested that in moderate patients, already at an early stage of the disease, a large scale of innate immune responses is activated, followed by the activation of adaptive immunity. In patients with severe symptoms, the innate immune responses are delayed, leading to the delayed priming of adaptive immune responses. This evidence encourages us to understand which metabolic factors can affect monocytes and macrophage programming in circulation and tissues since macrophages preferably activate glycolysis in acute inflammation, whereas homeostatically-balanced tissue macrophages privilege fatty acid metabolism pathways.

Two studies conducted a deep analysis of metabolic changes in the blood of patients with acute COVID-19, finding striking metabolic factors that correlate with the severity of the disease. The study by Martínez-Gómez et al. reported a multi-center, cross-sectional study examining the profile of amino acids and acylcarnitines in the serum of 453 COVID-19 patients using electrospray ionization–triple quadrupole tandem mass spectrometry. The authors used logistic models adjusted by age, sex, nutritional status, and comorbidities such as type 2 diabetes mellitus and hypertension. The authors found the strongest association of disease severity with circulating phenylalanine levels. Phenylalanine is a precursor of tyrosine and dopamine-related neurotransmitters that can potentially provide a connection to neurological symptoms in COVID-19 patients. To explain the increased levels of circulating phenylalanine, the authors offer a well-justified hypothesis of impaired hydroxylation and transamination caused by possible deficiency of co-factors such as BH4, NADH, oxygen, and vitamin B6. This hypothesis is supported by decreased citrulline levels, a metabolic derivate of arginine that also requires BH4, NADH, and oxygen. Loss of BH4 in chronic inflammatory conditions can reduce the biosynthesis of catecholamines, which consequently can disturb adrenergic neurotransmitter pathways in patients with neuropsychiatric symptoms like mood changes and depression (3). Thus hypoxia, resulting from impaired lung functions during acute SARS-CoV-2 infection, can be critical for the systemic amino-acid metabolism linked to neurological symptoms. Martínez-Gómez et al. also found the metabolic factors that provide a direct link to inflammation. These elements are an increase in the acetylcarnitine and 3-hydroxybutyryl/malonyl carnitine correlating with acute disease severity. This finding supports the body of evidence suggesting that COVID-19 patients over-utilize lipid beta-oxidation pathways due to the high energy demand provoked by the infection (4).

Ceballos et al. performed another metabolic plasma profiling by conducting a retrospective cross-sectional study enrolling 123 COVID-19 patients with asymptomatic/mild, moderate, and severe COVID-19. The authors applied untargeted-plasma metabolic profiling by gas chromatography and capillary electrophoresis-mass spectrometry, finding that metabolic plasma profiles differed from patients with severe or moderate disease at hospital admission. Moreover, the authors found that pro-inflammatory biomarkers significantly correlated with deregulated metabolites, particularly with L-kynurenine and L-tryptophan. The striking fact was the identification of gender-specific dysregulation of metabolites, cytokines, and chemokines between severely and moderately ill patients.

Chen et al. applied a weighted gene co-expression network (WGCNA) algorithm to analyze data from four peripheral blood transcriptomic datasets of COVID-19 patients, identifying essential genes for mild, moderate, and severe COVID-19. These genes were used as input for the next step of bioinformatic analysis. In this second step, the authors performed Short Time-series Expression Miner (STEM) analysis in a time-consecutive ischemia-reperfusion injury (IRI)-kidney dataset to search for the genes associated with renal injury in COVID-19. In severe COVID-19 patients, the research group found a limited group of genes related to the progression of renal injury, and a substantial part of these genes were regulators of immunometabolism. Of particular interest are TLR2 and TLR4, which serve as receptors for the unwanted-self pathogenic ligands that recognize and mediate macrophage inflammatory responses to the endogenous ligands elevated in diabetic conditions, where diabetic nephropathy is one of the major microvascular complications (5).

The immunometabolic mechanisms are essential due to their potential contribution to acute COVID-19 and as a crucial factor for the risk of long-COVID-19 diseases. Macrophages are well-known not only as targets for and first-line innate immune responses to viruses but also as the reservoirs of a large spectrum of viruses, including HIV, EBV, CMV, and influenza, that can replicate in macrophages (6). In our Research Topic, Matveeva et al. systematically organized the available data about SARS-CoV-2 entry pathways in macrophages, highlighting that virus-specific antibodies could enhance the entry of SARS-CoV-2 into macrophages, which is not necessarily enhancing the clearance of the virus. Several scenarios for the fate of the virus after its entry into macrophage are possible, where several studies already support abortive infection with a massive inflammatory cascade. However, other scenarios for long-term virus survival in macrophages, its reactivation leading to the production phase, and re-infection of permissive cells in the microenvironment are also possible. Evidence for such a scenario will come further. The challenging question for future research is which metabolic factors or conditions can re-activate resting SARS-CoV-2 in macrophages and which preferential organs could be affected.

The meta-analysis performed by Yang et al. in this Research Topic harvested evidence for the safety and efficiency of umbilical cord mesenchymal stromal cells (UC-MSCs) for the treatment of acute COVID-19. These data make us propose that UC-MSCs’ therapeutic effects are due to the control of the metabolism of resident tissue macrophages. However, such hypotheses need experimental confirmation in the future.

Surprising for respiratory viral infection and making the COVID-19 pandemic even more challenging were the neurological symptoms affecting the neuronal system’s sensory and cognitive functions, provoking conditions such as dementia (7). Identifying the metabolic crossroads between the viral life cycle and a unique profile of neurological symptoms is particularly interesting, especially considering the long-term consequences of SARS-CoV2 infection. In this sense, Matveeva et al. studied that COVID-19-related long-term neurological symptoms can be caused by the memory established during the acute illness and due to the potential long-term residence of SARS-CoV2 in the innate immune cells. The study of Fu et al. addressed the possible mechanism of Alzheimer’s disease (AD)-like cognitive impairment, a reported complication of SARS-CoV-2 infection. The authors investigated the diversity of the affected cells and pathways by searching the similarity between COVID-19 and Alzheimer’s disease using snRNA-seq and AD high-risk genes data. Of particular interest are the results obtained for microglia/macrophages. Analysis of microglia identified three markers, apolipoprotein E (APOE), membrane-spanning 4-domain subfamily A member 4A (MS4A4A), and protein-tyrosine kinase 2-beta (PTK2B), to have a common trend in patients with AD and COVID-19 compared to the control group, defined according to the AD-high-risk score. Most striking was the enhanced expression of PTK2B. PTK2B (also known as FAK2, PYK2, and RAFTK) is a non-receptor protein-tyrosine kinase that regulates the reorganization of the actin cytoskeleton, cell polarization, cell migration, and adhesion. Previous data informed that Pyk2-/- macrophages from a murine knock-out model fail in the polarization, membrane ruffling, and their ability to respond to chemokines that recruit them to sites of inflammation (8). This macrophage impairment was linked to compromised chemokine stimulation of inositol (1, 4, 5) triphosphate production and Ca2+ release, as well as integrin-induced activation of Rho and phosphatidyl inositol 3 kinases. ROS, the mediator of essential and primary macrophage response to infection, can stimulate PTK2B activity. We can speculate that overexpression of PTK2B in COVID-19 patients’ microglia is the leading mechanism for developing AD-like symptoms. In this context, elevated levels of Apo-E can additionally amplify local inflammation in the brain.

A few days ago, the World Health Organization (WHO) declared an end to COVID-19 global health emergency. Even though the pandemic is now a downward trend, we will continue seeing severe COVID-19 cases and mini-waves, as recently occurred in India with the SARS-CoV-2 variant XBB.1.16 (**9**). For this reason, we firmly believe that understanding immunometabolic mechanisms leading to robust inflammatory innate responses, sex-specific dysregulation of metabolites, macrophage overactivation, and neurological disorders is still crucial to identifying patients at higher risk of developing severe COVID-19 or long-term COVID-19 sequelae.

## Author contributions

All authors listed have made a substantial, direct, and intellectual contribution to the work and approved it for publication.
